# Therapeutic Targeting of Cancer Stem Cells via Modulation of the Renin-Angiotensin System

**DOI:** 10.3389/fonc.2019.00745

**Published:** 2019-08-08

**Authors:** Imogen M. Roth, Agadha C. Wickremesekera, Susrutha K. Wickremesekera, Paul F. Davis, Swee T. Tan

**Affiliations:** ^1^Gillies McIndoe Research Institute, Wellington, New Zealand; ^2^Department of Neurosurgery, Wellington Regional Hospital, Wellington, New Zealand; ^3^Upper Gastrointestinal, Hepatobiliary and Pancreatic Section, Department of General Surgery, Wellington Regional Hospital, Wellington, New Zealand; ^4^Wellington Regional Plastic, Maxillofacial and Burns Unit, Hutt Hospital, Wellington, New Zealand

**Keywords:** cancer stem cells, renin-angiotensin system, stem cell differentiation, tumorigenesis, bypass loops

## Abstract

Cancer stem cells (CSCs) are proposed to be the cells that initiate tumorigenesis and maintain tumor development due to their self-renewal and multipotency properties. CSCs have been identified in many cancer types and are thought to be responsible for treatment resistance, metastasis, and recurrence. As such, targeting CSCs specifically should result in durable cancer treatment. One potential option for targeting CSCs is by manipulation of the renin-angiotensin system (RAS) and pathways that converge on the RAS with numerous inexpensive medications currently in common clinical use. In addition to its crucial role in cardiovascular and body fluid homeostasis, the RAS is vital for stem cell maintenance and differentiation and plays a role in tumorigenesis and cancer prevention, suggesting that these roles may converge and result in modulation of CSC function by the RAS. In support of this, components of the RAS have been shown to be expressed in many cancer types and have been more recently localized to the CSCs in some tumors. Given these roles of the RAS in tumor development, clinical trials using RAS inhibitors either singly or in combination with other therapies are underway in different cancer types. This review outlines the roles of the RAS, with respect to CSCs, and suggests that the presence of components of the RAS in CSCs could offer an avenue for therapeutic targeting using RAS modulators. Due to the nature of the RAS and its crosstalk with numerous other signaling pathways, a systems approach using traditional RAS inhibitors in combination with inhibitors of bypass loops of the RAS and other signaling pathways that converge on the RAS may offer a novel therapeutic approach to cancer treatment.

## Cancer Stem Cells

As in normal tissue, tumors consist of diverse cell populations. The cellular heterogeneity observed in tumors has led to the suggestion that cancer may be sustained by cancer stem cells (CSCs), which, like normal embryonic stem cells (ESCs), are able to self-renew and undergo differentiation into multiple cell types. This is supported by several observations in cancer biology, including that only some tumor cells can recapitulate a tumor when xenografted into immunodeficient mice, and that tumors grown from tumorigenic cells consist of a mixed population of both tumorigenic and non-tumorigenic cancer cells (1). CSCs are thought to arise from either resident adult stem cells which have acquired oncogenic mutations or from progenitor cells which have an unlimited ability to replicate. CSCs share the properties of differentiation, self-renewal and homeostatic control with normal stem cells (1), express stem cell markers (2), and have subverted self-renewal pathways of normal stem cells (3).

The CSC concept proposes that cancer develops from a small subset of cells which can generate all the heterogeneous cell types seen within the tumor, including generating more CSCs as well as differentiated cancer cells. It has been shown in numerous tumor types that expression of certain markers can define populations of cancer cells which are able to generate a tumor, as well as their ability to respond to or resist cancer therapies, suggesting CSCs are present within these tumors (4). Studies transplanting mouse tumors into compatible wild-type mice have also shown that the cancer cells differ in their tumorigenic capacity, as only a small population of cancer cells are able to form tumors (4).

Given that adult human stem cells themselves are a diverse pool of cells expressing different markers, it is unsurprising that CSCs are also mixed populations of cells and are phenotypically and functionally diverse, and that the same tumor can contain multiple pools of CSCs (5). CSC diversity has also resulted in the emergence of a hierarchy, with a slow-cycling pool of cells giving rise to both a rapidly cycling population and non-proliferative cells, suggesting that targeting the cells with the potential to produce multiple types of tumor cells would be a beneficial approach to cancer treatment (6, 7). Heterogeneity within CSCs extends beyond tumorigenic potential and encompasses genetic and epigenetic changes as well as local environmental determinants and temporal and spatial differences (8). These differences have implications for effective therapies, as some cancer cells have been shown to resist chemotherapy and radiotherapy, and it has been suggested that they could be specifically targeted for differentiation as a therapeutic approach (8). Importantly, there is a level of plasticity within this system, as differentiated non-tumorigenic cancer cells can revert to CSCs (9, 10). This could be advanced by changes in the local environment driven by cues including hypoxia and inflammatory mediators to induce epithelial-mesenchymal transition and de-differentiation to increase the “stemness” of the tumor (5). This heterogeneity conferred by plasticity can result in treatment resistance (11).

CSCs have been shown to be capable of surviving radiotherapy and chemotherapy, which have no effect on the ability of the CSCs to regrow tumors (12). This resistance to radiotherapy is thought to occur by several mechanisms, including activation of DNA repair mechanisms, through activation of Wnt/β-catenin signaling, reactive oxygen species generation, and activation of other pro-survival signaling pathways (12). Resistance to chemotherapeutic agents is thought to occur via the use of drug efflux pumps and the expression of metabolic mediators (12). In addition, the quiescent, slow-cycling nature of CSCs is also likely to confer resistance to conventional treatments such as chemotherapy and radiotherapy which target rapidly dividing cells. The ability of CSCs to resist conventional cancer treatments has been well documented in breast cancer. Irradiation of mouse mammary primary epithelial cells enriches for progenitor cells (13), and breast cancer cells from patients following neoadjuvant chemotherapy are enriched for self-renewing cells (14). Furthermore, the number of CSCs and their ability to form mammospheres in culture is increased following chemotherapy of breast cancer patients (15) and Trastuzumab treatment of a breast cancer cell line (16).

Given their ability to generate a diverse cell population within a tumor and their ability to resist conventional cancer treatments, CSCs are proposed to be the cause of loco-regional recurrence and distant metastasis, and consequently treatment failure. This has implications for cancer therapy and suggests that the CSCs should be targeted for effective and durable cancer treatment. Consequently, several treatments targeting CSCs are currently in use in the clinic, with the main strategies being inhibiting key signaling pathways or directly targeting CSCs (17). These therapies include targeting CSC markers, such as CD44 and CD133, which have shown promise in a pre-clinical setting and therapies targeting these markers are in current clinical trials for acute myeloid leukemia and recurrent solid tumors, including liver, brain, pancreatic, breast, and colorectal cancers (18). In addition, a vaccination-based strategy against CSCs is in clinical trials for glioblastoma and other brain tumors (18), demonstrating the diverse approaches taken to target these cells. Given that CSCs express a unique set of markers, another approach toward identifying and eliminating these cells is to characterize other common features of CSCs and exploit these features for therapeutic targeting using drugs in common use, such as via modulation of signaling pathways such as the renin-angiotensin system (RAS).

## The Renin-Angiotensin System

### Physiological Control of Blood Pressure and Fluid Balance

**Figure 1:**
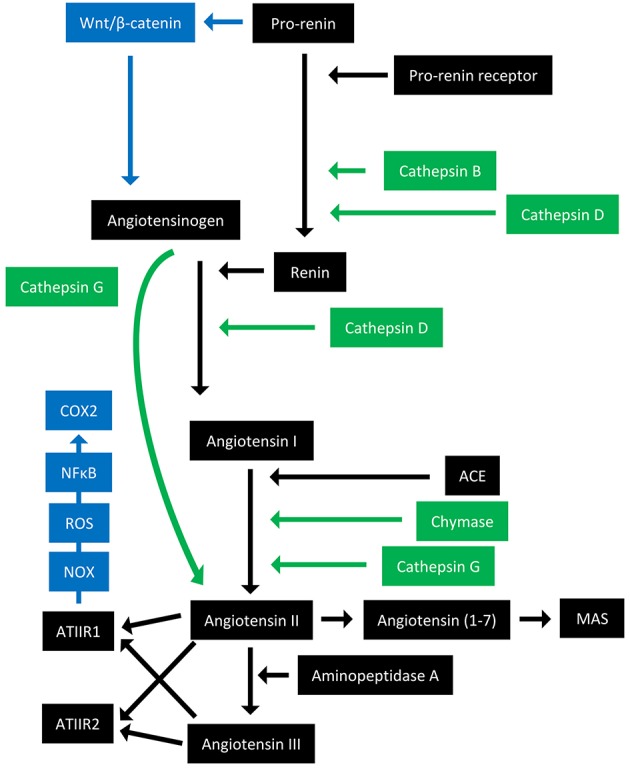
Overview of the renin-angiotensin system with its bypass loops and convergent signaling pathways. The renin-angiotensin system (black) regulates blood pressure, stem cell differentiation, and tumor development. Bypass loops of the RAS involving enzymes such as chymase and cathepsins B, D, and G (green) provide redundancy, while convergent inflammatory and developmental signaling pathways (blue) have multiple roles and effects. Angiotensinogen (AGN) is physiologically synthesized and released by the liver and is cleaved by renin to form angiotensin I (ATI). Renin is formed following binding of pro-renin to the pro-renin receptor. ATI is converted to angiotensin II (ATII) by angiotensin converting enzyme (ACE). ATII interacts with the G-protein coupled receptors ATII receptor 1 (ATIIR1) and ATII receptor 2 (ATIIR2) to restore homeostasis, via vasoconstriction and vasodilation, respectively. ATII can also give rise to angiotensin III via the action of aminopeptidase A, and Angiotensin 1–7 which binds and activates the G-protein coupled receptor MAS. Cathepsins B and D are also renin-activating enzymes that convert pro-renin to renin. Cathepsin D converts AGN to ATI, and cathepsin G converts ATI to ATII or AGN directly to ATII. Chymase converts ATI to ATII. Pro-renin also induces Wnt/β-catenin signaling in a feedback loop. ATIIR1 can also result in inflammatory signaling via the NOX-ROS-NFκB-COX2 signaling axis. ROS, reactive oxygen species.

**Figure 2:**
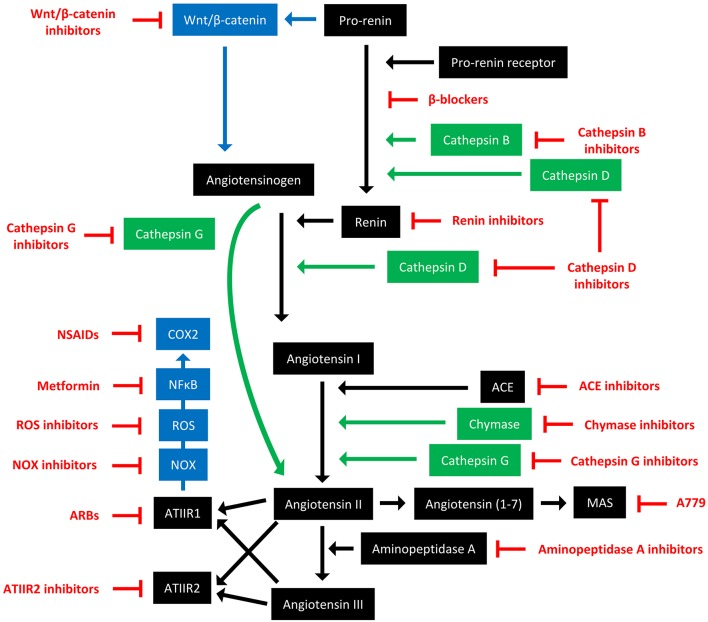
The renin-angiotensin system and its bypass loops and converging signaling pathways can be targeted at different points. The renin-angiotensin system (black) regulates blood pressure, stem cell differentiation, and tumor development. Bypass loops in the system involving cathepsins and chymase (green) provide redundancy, while convergent inflammatory and development signaling pathways (blue) have multiple roles and effects. Multiple points of the pathway can be targeted by specific inhibitors (red). ACE, angiotensin converting enzyme; ARBs, ATIIR1 blockers; ROS, reactive oxygen species; NSAIDS, non-steroidal anti-inflammatory drugs.

The RAS is an endocrine system crucial for the maintenance of homeostasis, as it regulates blood pressure and fluid balance via a signaling network (Figure 1). Physiologically, the RAS is activated in response to either reduced blood volume or blood pressure, and acts to restore homeostasis through the release of renin from the kidneys. Pro-renin is converted to active renin by binding to the pro-renin receptor (PRR). Renin then cleaves angiotensinogen, which is normally synthesized and released by the liver, giving rise to angiotensin I (ATI). ATI is then converted to angiotensin II (ATII) by angiotensin converting enzyme (ACE). Aminopeptidase A converts ATII to angiotensin III, and together they act on ATII receptors 1 and 2 (ATIIR1 and ATIIR2). These receptors have divergent actions, with ATIIR1 driving vasoconstriction and inhibiting renin to restore blood pressure, and ATIIR2 acting to promote vasodilation. Angiotensin 1-7 (Ang1-7) is the cleavage product of ATII and affects cardiovascular functions by binding to the G-protein coupled receptor MAS. However, there is considerable redundancy in the pathway with bypass loops involving proteases such as cathepsins B, D, and G, and the convergence of other signaling pathways on the RAS itself, including inflammatory pathways and Wnt/β-catenin signaling (Figure 1). Given the importance of the RAS for maintaining blood pressure, numerous modulators that inhibit the RAS at different points in the pathway have been developed (Figure 2). These groups of RAS inhibitors are commonly used in the clinic for the treatment of hypertension and include β-blockers, ACE inhibitors (ACEI), and ATIIR1 blockers (ARBs) as well as newer agents targeting other points in the pathway (e.g., renin inhibitors, chymase inhibitors, ATIIR2 inhibitors), inhibitors targeting bypass loops in the RAS pathway (e.g., cathepsin inhibitors), and inhibitors used in other canonical signaling pathways that converge on the RAS (e.g., Wnt/β-catenin inhibitors, metformin, and non-steroidal anti-inflammatory drugs) (Figure 2).

### Stem Cell Differentiation

Alongside its crucial role in fluid volume regulation, the RAS is also important for stem cell maintenance and differentiation in several cell types. ATII expression drives the differentiation of mesenchymal stem cells into adipocytes (19), while other components of the RAS drive differentiation into insulin producing cells (20). ACE is required for hemangioblast expansion, and modulation of ATIIR1 or ATIIR2 signaling can direct the fate of the blasts toward either an endothelial or hematopoietic lineage (21). The RAS also plays a role in hematopoiesis (22, 23), vasculogenesis (24), erythropoiesis (25, 26), and myeloid differentiation (27).

Importantly, the RAS not only acts to promote stem cell differentiation in diverse cell populations, but also appears to act in a feedback loop with Wnt/β-catenin signaling, where pro-renin receptor (PRR) can induce Wnt/β-catenin (28), and components of the RAS themselves are targets of Wnt/β-catenin (29). Wnt/β-catenin signaling is crucial for embryonic development and induces differentiation of pluripotent stem cells into progenitor cells (30). Given that Wnt signaling is also involved in cancer development (31), and downstream Wnt targets include the CSC markers CD44 and c-Myc (32), it may be that CSCs require activation of Wnt signaling (33). This suggests that RAS modulators could be employed in these cells to indirectly inhibit Wnt signaling and its effects.

Having identified these roles in normal stem cell maintenance and differentiation and feedback loops with a canonical developmental signaling pathway, it may be that the expression of the RAS also plays a role in the regulation or function of CSCs.

### Retrospective Studies and Clinical Trials Indicate Potential Benefit of RAS Modulators in Reducing Cancer Risk

The widespread use of RAS modulators as anti-hypertensives and their potential effect on cancer risk have been extensively documented. A seminal study has shown that the use of ACEI and ARBs is associated with a reduced risk of developing some cancer types, particularly cancers affecting women (34). Many other retrospective population studies have reported differing effects on cancer risk depending on the cancer type, cohort characteristics, and the RAS inhibitor used. As a result, several meta-analyses have been undertaken (35), again with differing results, which could be due to the nature of the original studies included and inherent publication bias. Aside from the reported effects on cancer risk, many retrospective population studies have also assessed the effect of RAS inhibitors on cancer death. Again, these results have been mixed, though meta-analyses have indicated that β-blocker use is not associated with survival in breast cancer patients (36), and a meta-analysis looking at ACEI use in all cancers showed no effect on cancer survival (37). A more recent meta-analysis looking at the use of different RAS inhibitors in all cancers showed that RAS inhibitor use extended overall, progression-free and disease-free survival (38). This is mainly due to ARBs and not ACEI use, with some site-specific effects. These studies need to be interpreted critically and with caution as they do not prove causality and the effect on cancer risk and mortality could be due to other factors. It may also be that a defined patient group will derive benefit from these treatments and that a more holistic approach of targeting the RAS in cancer is required to achieve a sustained treatment for patients. Due to the nature of the RAS, with its inherent bypass loops conferring redundancies, and the presence of many other pathways that converge on the RAS, it is likely that a multi-faceted approach to target the RAS will be required for effective cancer treatment.

Despite these disparate observations in retrospective population studies, the data around the involvement of the RAS in tumor models is clear, leading to many clinical trials using RAS inhibitors and the development of new targeted agents (39–41). Several of these studies have trialed ARBs in cancer patients, with Losartan being shown to enhance the efficacy of chemotherapy and improve overall survival in ovarian cancer patients (42). Another ARB, Candesartan, has been shown to decrease prostate specific antigen levels in hormone-refractory prostate cancer patients (43), and is tolerated in advanced pancreatic cancer (44, 45). The ACEI Captopril is tolerated in patients with advanced cancer (46), and has been shown to reduce biochemical recurrence in prostate cancer patients (47), while Perindopril reduced the risk of recurrence of hepatocellular carcinoma as a combination therapy with other non-traditional treatments (48, 49). Several trials have targeted the Ang1-7/MAS axis in breast cancer before or after chemotherapy (50), and in metastatic sarcoma, where it is well tolerated (51), and a number of advanced solid tumors where it provides benefit for some patients (52).

β-blockers work by blocking β-adrenergic receptors to prevent neurotransmitter binding. This prevents renin secretion and its actions and subsequently results in lowered blood pressure (Figure 2). The non-selective β-blocker Propranolol has been shown in several case reports to be efficacious in treating angiosarcoma (53), and in combination with chemotherapy treatment induced responses in seven patients with advanced angiosarcoma (54). Another study showed that addition of Propranolol or another non-selective β-blocker Carvedilol to treatment regimens for metastatic angiosarcoma improved progression-free and overall survival (55). Propranolol has also been used in a proof of concept study in multiple myeloma patients receiving hematopoietic cell transplantations (56), and in a prospective cohort study in melanoma patients where its use was associated with reduced recurrence (57).

While these trials have demonstrated promise for targeting the RAS in cancer treatment, the mechanisms by which this is achieved are yet to be elucidated. Current clinical trials and the development of new RAS targets should help to further define which patient groups may benefit from these treatments.

### *In vitro* and *in vivo* Cancer Models Rationalize the RAS as a Therapeutic Target

Given the potential effects on reducing cancer risk observed in retrospective population studies, expression of components of the RAS have been assessed in many different tumor types to clarify the potential role of the RAS in tumorigenesis (Table 1).

**Table 1:** Components of the RAS are expressed in tumors.

**RAS component**	**Expression in tissue**	**Tumor types and references**
Pro-renin receptor	Increased expression	Endometrial cancer (58)
Angiotensinogen	Increased expression	Lung cancer (59)
ACE	Increased expression	Prostate cancer (60), gastric cancer (61), endometrial cancer (58)
	Polymorphism correlated with metastases	Gastric cancer (62)
ATIIR1	Deficiency reduces tumor growth and angiogenesis	Melanoma (63), sarcoma (64), lung cancer (65), fibrosarcoma (66)
	Increased expression	Pancreatic cancer (67), ovarian cancer (68), prostate cancer (60), astrocytoma (69), breast cancer (70), renal clear cell carcinoma (71)
	Expression associated with disease progression	Ovarian cancer (68)
	Expression associated with poor survival	Intestinal type gastric cancer (72), astrocytoma (69)
ATIIR2	Deficiency increases tumor growth	Pancreatic cancer (73)
	Increased expression	Gastric cancer (61), endometrial cancer (58)
	Reduced expression	Lung cancer (59)
	Expression associated with poor survival	Astrocytoma (69), renal clear cell carcinoma (71)
Cathepsin B	Expression associated with poor survival	Gastric cancer (74)
Cathepsin D	Increased expression	Hepatocarcinoma (75), melanoma (76), colorectal cancer (77), prostate cancer (78)
	Expression increases metastasis	Liver metastases (79, 80)
	Expression associated with poor survival	Breast cancer (81–84)

These studies have helped define the role of the RAS in tumorigenesis, and collectively show that components of the RAS are expressed in many different cancer types (39, 85). The effects on tumor growth, angiogenesis, metastasis and survival indicate that the RAS plays a role in the hallmarks of cancer (39, 86, 87). It is also thought to contribute to an immunosuppressive microenvironment in tumors and reduce infiltration of tumor-associated macrophages (88). The increased expression of components of the RAS in different cancer types may contribute to tumorigenesis and the poor clinical outcome seen in some cancer types. This suggests that regulation of the RAS may be a general mechanism for cancer prevention and warrants further investigation to understand the precise underlying mechanisms.

Given that the RAS is over-expressed in many cancer types and the use of RAS modulators may affect cancer risk and cancer survival, numerous studies have assessed the effect of RAS inhibitors *in vitro* and on tumor models *in vivo*. These have focused on β-blockers (Table 2), ACEI (Table 3), and ARBs (Table 4) to assess the role of the RAS in tumor development.

**Table 2:** β-blockers inhibit tumorigenesis in cell and animal models.

**Drug name**	**Effect in tumor models or cell lines**	**Tumor types and references**
Propranolol	Inhibition of growth and proliferation	Pancreatic ductal adenocarcinoma (89), breast cancer (90, 91), neuroblastoma (92), angiosarcoma (55, 93), melanoma (94–97), pancreatic cancer cells (98), gastric cancer cells (99, 100), neuroblastoma cells (92), hemangioendothelioma cells (93), angiosarcoma cells (55, 93), colorectal cancer cells (101), melanoma cells (94, 96), breast cancer cells (102), liver cancer cells (103), prostate cancer cells (104)
	Inhibition of migration	Colon carcinoma cells (105), breast cancer cells (106)
	Inhibition of invasion	Ovarian cancer cells (107), pancreatic cancer cells (108)
	Inhibition of metastasis	Prostate cancer (109), melanoma (95)
	Prolonged survival of tumor-bearing animals	Neuroblastoma (92)
Carvedilol	Inhibition of growth and proliferation	Neuroblastoma and neuroblastoma cells (92)
Nebivolol	Inhibition of growth and proliferation	Neuroblastoma and neuroblastoma cells (92)

**Table 3:** ACE inhibitors inhibit tumorigenesis in cell and animal models.

**Drug name**	**Effect in tumor models or cell lines**	**Tumor types and references**
Captopril	Reduced growth	Renal cancer (110), lung cancer (111), colorectal cancer liver metastases (112, 113), lung cancer cells (111), esophageal squamous cell carcinoma cells (114)
	Increased growth	Fibrosarcoma (115)
	Reduced metastases	Lung cancer (111)
	Decreased survival of tumor-bearing animals	Renal cancer (115)
Enalapril	Inhibition of growth	Pancreatic cancer (116, 117), neuroendocrine cancer cells (117)
	Inhibition of invasion	Pancreatic cancer (116), gastric cancer cells (61)
Perindopril	Reduced growth and angiogenesis	Hepatocellular carcinoma (118–120)

**Table 4:** ARBs inhibit tumorigenesis in cell and animal models.

**Drug name**	**Effect in tumor models or cell lines**	**Tumor types and references**
Candesartan	Inhibition of growth and proliferation	Gastric cancer cells (121), lung cancer cells (122)
	Reduced angiogenesis	Renal cancer (123), ovarian cancer (68), breast cancer (124)
	Reduced metastases	Renal cancer (123)
	Prolonged survival of tumor-bearing animals	Peritoneal carcinomatosis (121)
Irbesartan	Reduced growth	Colorectal cancer liver metastases (112), esophageal squamous cell carcinoma cells (114)
Losartan	Reduced growth	Breast cancer (70), esophageal squamous cell carcinoma cells (114)
	Increased proliferation	Melanoma cells (125)
	Reduced invasion	Breast cancer (70)
	Reduced angiogenesis	Pancreatic cancer (126)
Olmesartan	Reduced invasion	Gastric cancer cells (61)
Telmisartan	Inhibition of growth and proliferation	Prostate cancer cells (127), uterine leiomyoma cells (128), lung cancer cells (129)

Studies investigating β-blockers in cancer (Table 2) have largely used the β-blocker Propranolol and have shown that across a wide range of cancer types, Propranolol inhibits the growth of tumors and tumor cells. This suggests that Propranolol could be repurposed for cancer treatment (130, 131), as has been the case for the benign vascular tumor infantile hemangioma for which it is an effective treatment (132–134).

Given the effects of β-blockers on cancer and cancer cell growth, other studies have investigated the impact of other classes of drugs that modulate the RAS on neoplastic processes. One of these classes is ACEIs, which block the action of ACE and hence downstream production of ATII (Figure 2). Studies looking at ACEIs (Table 3) are extensive and demonstrate that this class of drugs (including Captopril, Enalapril, and Perindopril) appear to prevent tumor growth and invasion in many different tumor types and models.

Another broad class of drugs that modulate the RAS are ARBs, which block ATIIR1 (Figure 2). Studies using ARBs to assess cancer development in cell and animal models (Table 4) have also shown that different drugs within this class (Candesartan, Irbesartan, Losartan, Olmesartan, and Telmisartan) inhibit tumor development across several tumor types.

These studies underscore the complex nature of the RAS and suggests that different RAS modulators may have different effects in different tumor types. Taken together, they suggest that anti-hypertensive drugs which target the RAS have shown promise for repurposing in the cancer setting. Across several classes of drugs (β-blockers, ACEIs, and ARBs) in both *in vitro* and *in vivo* models, they have been shown to reduce tumor cell growth, migration, invasion, and metastasis in numerous cancer types. These processes comprise many of the characteristics of CSCs and of the hallmarks of cancer (135), and are consistent with the expression of some components of the RAS in high-grade disease and the associated poor survival (Table 1). This suggests that there is merit in repurposing RAS inhibitors for cancer treatment. Many clinical trials using this approach are currently underway, despite limited functional work and mechanistic understanding about how this approach might work in cancer patients. With the development of new agents targeting specific parts of the pathway, including the bypass loops, and the refinement of existing drugs, new opportunities are emerging for modulating the RAS pathway, either in combination with current therapies or by targeting the entire RAS and its bypass loops, and pathways converging on the RAS.

## Cancer Stem Cells Express Components of the RAS

Given the well characterized role of the RAS in both stem cell maintenance and tumorigenesis, it is possible that these functions are directed by RAS signaling in CSCs. In order to demonstrate this, it is important to first establish that CSCs express both CSC markers and components of the RAS. This has been shown to be the case in numerous cancer types, including glioblastoma (136–138), metastases to the liver from colon adenocarcinoma (139, 140), head and neck cutaneous squamous cell carcinoma (141), and oral cavity squamous cell carcinoma affecting the buccal mucosa (142, 143), oral tongue (144–146), and lip (147, 148). In addition, components of the RAS have also been demonstrated on the tumor stem cells of benign tumors such as meningioma (149, 150), infantile hemangioma (151, 152), and pyogenic granuloma (153). Importantly, the expression of cathepsins B, D, and G in some of these cancer types (74, 138, 140, 146, 150) suggests the presence of bypass loops of the RAS which could circumvent the action of traditional RAS inhibitors and offer a potential explanation for the differing findings of cancer risk and cancer survival with long-term use of traditional RAS inhibitors. Given the presence of components of the RAS in CSCs in these cancers, it is possible that the expression of these components is controlling the differentiation and function of the CSCs within these tumors.

Despite the indirect evidence from retrospective population studies and more substantial direct evidence from *in vitro* studies and *in vivo* tumor models, very little is known about the mechanism by which RAS modulators influence tumor development. Although expression of components of the RAS has been demonstrated in CSCs, their function and how they might respond to RAS modulators has yet to be characterized. However, the fact that many clinical trials involving targeting of the RAS in cancer have taken place and are currently underway underscores the role of RAS in tumorigenesis and the need for further investigations into this system. Importantly, the findings in tumor model systems are seen consistently across a broad range of tumor types, suggesting its common role in cancer biology which may be affected through CSCs and their functions.

The expression of both components of the RAS and CSC markers in several cancer types may indicate that the CSCs may be a novel therapeutic target through modulation of the RAS. It is possible that a multi-faceted strategy simultaneously targeting multiple critical points of the RAS and related signaling pathways may result in durable cancer treatments by altering CSC function. Indeed, Phase II trials in metastatic renal cell carcinoma using either Perindopril or Candesartan in combination with other agents, including a cyclooxygenase-2 inhibitor have shown potential for stabilizing the disease and reducing recurrence (154). Propranolol treatment in combination with a cyclooxygenase-2 inhibitor is well tolerated in breast cancer patients and transcriptional profiling showed the combination reduced markers of invasion and inflammation (155). Furthermore, targeting other pathways which converge on the RAS may also prove worthwhile, as Metformin selectively kills CSCs in mouse breast cancer models (156), and targeting Wnt signaling is known strategy for CSC elimination (157, 158).

## Conclusion

The involvement of the RAS in both tumor development and stem cell maintenance suggests that these roles may converge on CSC maintenance and function. Given the ability of CSCs to promote cell migration, invasion and metastasis (17), and the reduction of these processes by RAS inhibitors *in vitro* and *in vivo*, it may be that the success of RAS inhibitors in reducing cancer risk and improving cancer survival is due to their effects on CSCs. In support of this, components of the RAS and enzymes that constitute bypass loops of the RAS have been shown to be expressed in CSCs of several different cancer types. This offers an avenue for targeted therapies using RAS inhibitors, modulators of the bypass loops, and agents targeting other signaling pathways that converge on the RAS. Importantly, RAS inhibitors are commonly available, well tolerated and inexpensive and have been shown to be effective in controlling tumor growth in several settings. However, many of these studies have relied on immortalized cancer cell lines and xenograft tumor models, and in order to better understand the mechanisms of these drugs and the discrepancies observed in their effects clinically, models closer to the patient need to be employed. In addition, the nature of the RAS and its crosstalk with other pathways means a system-wide approach simultaneously targeting multiple key steps of the RAS is needed to achieve effective cancer control.

## Author Contributions

IR drafted the manuscript. AW, SW, PD, and ST commented on the manuscript. All authors approved the manuscript.

### Conflict of Interest Statement

PD and ST are inventors of the of the patent Cancer Stem Cells (US15/503025) and the PCT patent Cancer Therapeutic (PCT/NZ2018/050006), and the provisional patent application Novel Pharmaceutical Compositions for Cancer Therapy (US/62/711709). The remaining authors declare that the research was conducted in the absence of any commercial or financial relationships that could be construed as a potential conflict of interest.
